# Characterization of three naturally occurring lignans, sesamol, sesamolin, and sesamin, as potent inhibitors of human cytochrome P450 46A1: Implications for treating excitatory neurotoxicity

**DOI:** 10.3389/fphar.2022.1046814

**Published:** 2022-11-22

**Authors:** Jie Du, Xiaodong Chen, Yongshun Zhao, Tingting Zhao, Dalong Wang, Zujia Chen, Changyuan Wang, Qiang Meng, Jialin Yao, Huijun Sun, Kexin Liu, Jingjing Wu

**Affiliations:** ^1^ Department of Clinical Pharmacology, College of Pharmacy, Dalian Medical University, Dalian, China; ^2^ Provincial Key Laboratory for Pharmacokinetics and Transport, Liaoning Dalian Medical University, Dalian, China; ^3^ Department of Neurosurgery, The First Affiliated Hospital, Dalian Medical University, Dalian, China

**Keywords:** sesame lignans, CYP46A1, 24S-hydroxycholesterol, NMDAR, excitatory neurotoxicity

## Abstract

CYP46A1 is a brain-specific enzyme responsible for cholesterol homeostasis. Inhibition of CYP46A1 activity serves as a therapeutic target for excitatory neurotoxicity. Sesame is a common medicine and food resource; its component lignans possess various pharmacological activities. In this study, the inhibitory effects of sesame lignans on CYP46A1 activity were investigated. Inhibition kinetics analyses revealed that sesamin and sesamolin produce mixed partial competitive inhibition of CYP46A1, while sesamol produces non-competitive inhibition. Notably, molecular simulations revealed that the sesame lignans have excellent orientations within the active cavity of CYP46A1. Importantly, the sesame lignans had high permeability coefficients and low efflux ratios. Furthermore, sesamin significantly reduced the levels of 24S-hydroxycholesterol in rat plasma and brain tissues, and down-regulated the protein expressions of CYP46A1, NMDAR2A, NMDAR2B, and HMGCR. Collectively, sesame lignans exhibit significant inhibitory effects on CYP46A1 activity, highlighting their potential therapeutic role in treating excitatory neurotoxicity.

## Introduction

Cholesterol 24S-hydroxylase (CYP46A1) is a brain-specific cytochrome P450 family enzyme encoded by CYP46A1. It converts cholesterol into 24S-hydroxycholesterol (24*S*-HC) and is the dominant cholesterol catabolic enzyme in the brain (Russell et al., 2009). Tissues acquire cholesterol from two sources; the first is an exogenous source in the form of plasma lipoproteins and the second is an endogenous source in the form of a biosynthetic pathway comprising over 20 enzymes that convert Acetyl-CoA into cholesterol. Since the blood-brain barrier prevents plasma lipoprotein particles from entering the brain, it relies entirely on cholesterol synthesis to meet its cholesterol needs (Jurevics and Morell, 1995). Regulation of the circulation of the neuronal cholesterol pool occurs primarily through CYP46A1, which converts cholesterol into 24*S*-HC; 24*S*-HC is eliminated by crossing the blood-brain barrier and entering the liver (Zlokovic, 2008). CYP46A1-mediated 24*S*-HC flux is important for the turnover of cholesterol in the brain and affects the basic properties of membranes and synaptic transmission (Moutinho et al., 2016). Thus, altered CYP46A1 activity affects brain physiology.

A growing body of evidence suggests that modulation of CYP46A1 could serve as a therapeutic drug target for the central nervous system as well as a basic research tool to investigate the function of CYP46A1 in the brain. Both inhibition and activation of CYP46A1 have therapeutic potential. Several research groups have reported that activation of CYP46A1 activity can ameliorate the symptoms of Alzheimer’s disease (AD) and Huntington’s disease (HD) in animals (Hudry et al., 2010; Chali et al., 2015; Djelti et al., 2015; Boussicault et al., 2016; Mast et al., 2017). Conversely, inhibition of CYP46A1 activity is hypothesized to reduce seizure frequency (Sun et al., 2016; Petrov and Pikuleva, 2019). Preclinical studies in mice suggest that soticlestat-mediated inhibition of CYP46A1 may have therapeutic potential for diseases associated with neural hyperexcitation. Soticlestat is currently being investigated as a drug for the treatment of Dravet syndrome and Lennox-Gastaut syndrome with a novel mechanism of action (Bialer et al., 2018; Strzelczyk and Schubert-Bast, 2020; Halford et al., 2021).

**FIGURE 1:**
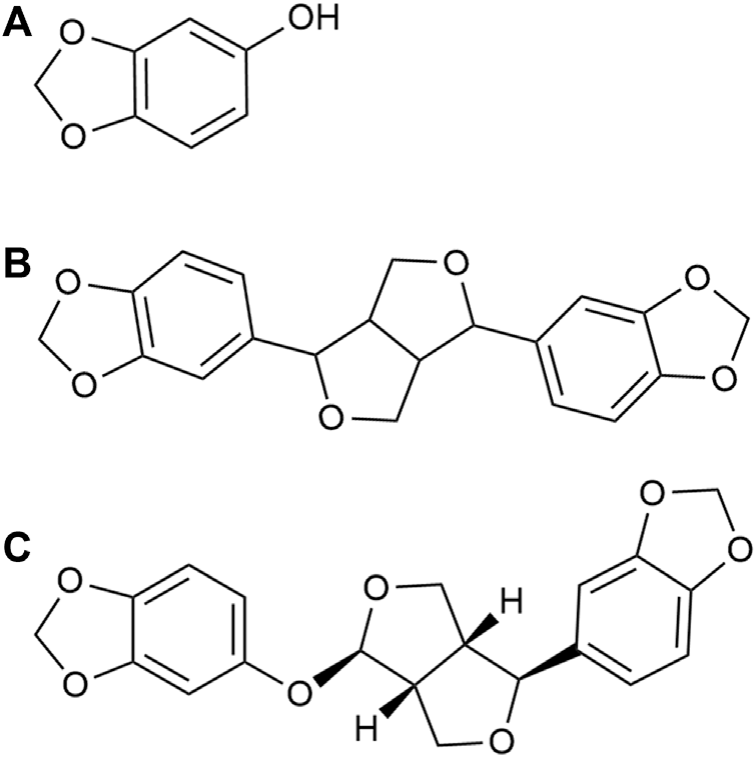
Chemical structures of **(A)** sesamol **(B)** sesamin, and **(C)** sesamolin.

Sesame, also known as Sesamum indicum, is an annual plant that is a member of a widely cultivated genus. Sesame is an important oilseed with edible and potential nutritional and medical uses in most countries (Morris et al., 2021). Sesame seeds have a variety of medicinal properties and are used for their tonic, nutritive, and diuretic properties in the treatment of asthma, dry cough, ulcers, inflammation, urinary diseases, vertigo, lung diseases, and migraines (Mili et al., 2021). Clinically important antioxidant sesame lignans, including sesamin, sesamolin, and sesamol, are obtained from Sesamum indicum (Dar and Arumugam, 2013) (Figure 1). These sesame lignans have been found to exhibit varied biological activities. Sesamin is reported to reduce the expression of lipogenic enzymes by binding to peroxisome proliferator-activator receptor alpha (PPAR-α) (Peñalvo et al., 2006). In addition, it is associated with decreased expression of sterol regulatory element binding protein-1 (SREBP-1), acetyl-CoA carboxylase, and fatty acid synthase, and can inhibit cholesterol absorption and biosynthesis in rats and humans (Hirose et al., 1991; Hirata et al., 1996; Takashi et al., 2003). Moreover, sesamin has a neuroprotective effect on cerebral ischemia injury in middle cerebral artery occlusion reperfusion rats; this effect is thought to be due to the inhibition of neurological impairments and oxidative damage, followed by the inhibition of apoptotic responses (Khan et al., 2010). Sesamin effectively reduced glutamate release and calcium influx by antagonizing glutamate receptors, thus alleviating excitatory neurotoxicity injury (Jiang et al., 2022). Sesamolin has been found to reduce reactive oxygen species (ROS) and inhibit apoptosis in neuron cells, providing neuroprotection against hypoxia-induced ROS and oxidative stress (Rosalina and Weerapreeyakul, 2021). Sesamol has also been shown to protect cell membranes against lipid peroxidation and to prevent low-density lipoprotein (LDL) oxidation and microsomal peroxidation (Dar and Arumugam, 2013). Sesamol also alleviates seizures and improves cognitive dysfunction and oxidative stress in a pentylenetetrazol-induced kindling model of epilepsy (Hassanzadeh et al., 2014). CYP46A1 is thought to be involved in excitatory toxicity; however, it remains unclear whether these sesame lignans interact with CYP46A1, thus explaining the neuroprotective properties of these lignans.

In our preliminary study, the inhibitory effects of sesamin, sesamolin, and sesamol on CYP46A1 were evaluated. Surprisingly, sesamin, sesamolin, and sesamol displayed significant inhibitory activity against CYP46A1. Therefore, we hypothesized that sesame has an effect on cholesterol metabolism in the brain by regulating the activity of CYP46A1, which, at least partly, explains its neuroprotective effect. Herein, we characterized the inhibitory kinetics of sesame lignans and explored the relationship between structure and inhibition. Moreover, the influence of sesamin on the activity and expression of CYP46A1 was investigated *in vivo*.

## Material and methods

### Chemicals and reagents

Testosterone and digoxin were obtained from Dalian Meilun Biotech Co., Ltd (Dalian, China). 4-androsten-16β,17β-diol-3-one (16β-hydroxytestosterone) was obtained from Steraloids, Inc. (Newport, Rhode Island, United States). Sesamin (purity >98%) was obtained from Tokyo Chemical Industry Co., Ltd. Sesamolin (purity >98%) was obtained from Shanghai Aladdin Bio-Chem Technology Co., Ltd (Shanghai, China). Sesamol (purity >98%) was purchased from Sigma-Aldrich (MO, United States). Nicotinamide adenine dinucleotide phosphate (NADPH) and Carbamazepine were acquired from Shanghai Yuanye Bio-Technology Co., Ltd (Shanghai, China). The MDCK-MDR1 cell line was generously donated by Professor Su Zeng, Zhejiang University (Zhejiang, China). Neuro-2a stable cell line overexpressed in human CYP46A1 (h-CYP46A1-Neuro-2a) was purchased from Hanbio Biotechnology Co., Ltd. The cDNA-expressed recombinant human cytochrome P450 (rhCYP) isoforms, including CYP46A1, derived from baculovirus-infected insect cells co-expressing NADPH-CYP reductase and cytochrome b5 were purchased from Cypex (Dundee, Scotland, United Kingdom). A BCA Protein Assay Kit was purchased from Beijing Solarbio Science and Technology Co., Ltd. Anti-Cholesterol-24 Hydroxylase antibody was obtained from Sigma-Aldrich (Darmstadt Germany). NMDAR2A antibody and NMDAR2B antibody were purchased from Proteintech Group (Wuhan, China). HMGCR Rabbit mAb was obtained from ABclonal Technology Co., Ltd. Analytical reagent grade and high-performance liquid chromatography (HPLC) grade solvents were obtained from Tedia, Inc. (OH, United States).

### Incubation conditions

16β-hydroxytestosterone formation catalyzed by CYP46A1 was conducted at conditions linear for the duration of incubation and protein concentration. The incubation mixture (100 μL) consisted of 0.5 μL testosterone (final concentration: 10 μM, relevant to the *K*_m_ value), 100 mM potassium phosphate buffer (pH 7.4), 4 μL recombinant human CYP46A1 bactosomes (final concentration: 0.4 mg/ml), and 0.5 μL sesame lignans. After preliminary incubation at 37°C for 5 min, 10 μL NADPH (final concentration, 1 mM) was added to initiate the reaction for 10 min. The reaction was stopped by adding the same volume of acetonitrile containing 1 μM of the internal standard. The mixture was left on ice until it was centrifuged at 20,000×g for 20 min at 4°C. Aliquots of the supernatant were stored at −20°C until analysis. The possible inhibitory intensities of the sesame lignans were evaluated by quantifying the 16-OH-TST generated.

### Analytical methods

Testosterone 16β-hydroxylation reaction samples were injected into an API 3200 triple quadrupole mass spectrometer (Applied Biosystems, Concord, Ontario, Canada) and an Agilent HP1200 LC system (Agilent Technology Inc., CA, United States). The chromatographic separation was performed on a Hypersil ODS-BP column (150 mm × 2.1 mm, 5 μm; Dalian Elite Analytical Instruments Co. Ltd., China) at ambient temperature. IonSpray Voltage (IS): 4500 V; Temperature (TEM): 590°C; Ion Source Gas1 (GS1, N_1_): 25 L/min; Ion Source Gas2 (GS2, N_2_): 35 L/min; Curtain Gas (CUR): 30 psi; Collision Gas (CAD): 12 psi. MS/MS conditions for analyzed compounds are shown in Supplementary Table S1. Mobile phase A consisted of 0.1% formic acid in water; mobile phase B consisted of 0.1% formic acid in acetonitrile; the flow rate was 0.4 ml/min. The gradient elution program was 0–1 min, 95% A; 1–3 min, 95%–65% A; 3–5 min, 65%–35% A; 5–6 min, 35%–15% A; 6–8 min, 15% A; 15%–95% A, 8–8.1 min; 8.1–12 min, 95% A. 16β-hydroxytestosterone and Carbamazepine (internal standard) were monitored in the positive ion mode, and detected by fragment ions at m/z 305.2→97.2 and 237.1→194.2, respectively. LC-MS/MS data acquisition and integration were performed with Analyst 1.6.3 using the internal standard and peak-area ratios for calculation (linear regression, 1/x weighting).

### Inhibition kinetics analysis

Under the above incubation conditions, different concentrations of sesame lignans at the same concentration of testosterone (10 μM) were first used to measure the IC_50_ values. *K*_i_ was determined at various concentrations of testosterone (2–150 μM) in the presence or absence of sesame lignans using Prism (Version 8.0.2, GraphPad, San Diego, CA). *K*_*i*_ values were calculated using the equations for competitive inhibition (Eq. 1), non-competitive inhibition (Eq. 2), or mixed inhibition (Eq. 3). The three equations and the inhibition constant were as described in our previously published methods (Wu et al., 2017).

$$v=\frac{V_{\max}\cdot S}{K_{m}\cdot \left(1+\frac{I}{K_{i}}\right)+S}$$ (1)

$$v=\frac{V_{\max}\cdot S}{\left(K_{m}+S\right)\cdot \left(1+\frac{I}{K_{i}}\right)}$$ (2)

$$v=\frac{V_{\max}\cdot S}{K_{m}\cdot \left(1+\frac{I}{K_{i}}\right)+S\cdot \left(1+\frac{I}{\alpha \cdot K_{i}}\right)}$$ (3)

where *v* is the reaction velocity; S and I denote the substrate and inhibitor concentrations, respectively; *K*_*i*_ represents the inhibition constant describing the affinity of the inhibitor for the enzyme; *K*_m_ refers to the substrate concentration at half-maximal velocity (*V*_max_) of the reaction. The *α* value determines the degree of change in enzyme affinity for the substrate following the binding of the inhibitor. When *α* is less than 1, binding of an inhibitor prevents substrate binding, and the mixed model is identical to non-competitive inhibition. When *α* is very large (*α* > 1), the binding of an inhibitor prevents substrate binding, and the mixed model depicts competitive inhibition. The type of inhibition was determined by fitting the data to enzyme inhibition models. The goodness-of-fit of kinetic and inhibition models was assessed using *R* ^2^ values and parameter S.D. estimates. Dixon and Lineweaver-Burk plots were also used to characterize the inhibition kinetics. Kinetic constants are reported as the mean ± S.D. of the parameter estimate.

### Docking simulation

To illustrate the potential interactions between the sesame lignans and CYP46A1, docking simulations were performed using the Sybyl/Surflex module. The structures of sesamin, sesamolin, and sesamol were downloaded from SciFinder. Based on the crystal structure of CYP46A1 (PDB ID: 2Q9F), the bioactive binding conformations of sesamin, sesamolin, and sesamol and soticlestat (a known specific CYP46A1 inhibitor) were evaluated using the empirical function Total Score. A 2D molecular descriptor (Discovery Studio Visualizer software, version 3.5) was used to show the interacting amino acid residue plot of the ligands located within the CYP46A1 active site. In addition, the docking results of sesamin, sesamolin, and sesamol were visualized using the PyMOL Molecular Graphics System, version 0.99 (DeLano Scientific LLC).

### Membrane permeability assay

To estimate the permeability of the sesamin lignans across the blood-brain barrier (BBB), a membrane permeability assay was conducted with an MDCK-MDR1 monolayer. The transport assays were performed by seeding MDCK-MDR1 cells on a tissue culture plate insert (BIOFIL^®^, PC membrane, 0.33 cm^2^ surface area, 0.4 μm pore size) in 24-well companion plates for 5 days. The culture medium was changed once every 2–3 days. To confirm the confluency of the cell monolayer, the trans-epithelial electrical resistance (TEER) of each well was measured before the transport assay using a resistance meter (Millipore, Millicell^®^ ERS-2, United States). The transport experiment was performed only on inserts with TEER values above 300 Ω cm^2^ for MDCK-MDR1 cells (Jin et al., 2020). The cell monolayer was gently washed with Hank’s Balanced Salt Solution (HBSS) at 37°C and the transwell chamber was pre-incubated for 30 min. Sesamin (20 μM), sesamolin (20 μM), sesamol (20 μM) and digoxin (5 μM) were added to the HBSS in the initial compartment, and drug-free HBSS was added to the receiver compartment. After 120 min of incubation, a 50 μL sample was removed from the receiver compartment and uniformly mixed with 50 μL methanol. Then, it was centrifuged at 20,000 ×g at 4°C for 20 min. All available samples in the receiver compartments were injected into the HPLC system for analysis. The HPLC system comprised an Agilent MSD/MS system controller, two 1,260 series pumps, a 1,200 series autosampler, a 1,200 series variable wavelength detector, and an elite ODS-BP analysis column (4.6 × 200 mm, 5 μm). The mobile phase for sesame lignans was methanol/water (80/20, v/v), and the flow rate was 0.8 ml/min with a detector wavelength of 290 nm. The mobile phase for digoxin was acetonitrile/water (32/68, v/v), and the flow rate was 0.8 ml/min. Ultraviolet detection was performed at 230 nm. The permeability (*P*_app_) and efflux ratio (ER) were calculated as follows (Eqs. 4, 5) (Grès et al., 1998).

$$P_{app}=\frac{dQ}{dt}\cdot \frac{1}{A\cdot C_{0}}$$ (4)

In Eq. (4), A denotes the area of mass transfer, C_0_ represents the donor concentration of compound in the upper medium, and dQ/dt is the rate of transmembrane transportation. When the *P*_app_ value is greater than 1 × 10^–6^ cm/s, the compound is considered to show high permeability. Conversely, when the *P*_app_ value is less than 1 × 10^–7^ cm/s, the compound has low permeability (Artursson et al., 2001).

$$ER=\frac{P_{app,\;B\to A}}{P_{app,\;A\to B}}$$ (5)

In Eq. (5), *P*_app, B→A_ denotes the basal to apical (B-A) transport rate, and *P*_app, A→B_ is the apical to basal (A-B) transport rate. When the ER value is greater than or equal to 2, the investigated drug is considered to be a substrate of the efflux transporter (e.g., P-gp) in cells that express P-gp, according to FDA guidelines (Food and Drug Administration, 2020).

### CCK-8 assay

First, The Neuro-2a-CYP46A1 cells were seeded into 96-well plates at a density of 1 × 10^4^ cells per well. When the cells had grown to approximately 80% of the plate, the complete medium was replaced with serum-free medium containing sesamol, sesamin, and sesamolin at indicated concentrations for 24 h. Then, serum-free medium containing 10 μL of CCK-8 solution was added to each well and the cells were incubated at 37°C. The absorbance was assessed at 450 nm with a microplate reader (Tecan, Austria).

### Cell culture and treatment

MDCK-MDR1 cells and Neuro-2a-CYP46A1 cells were cultured in DMEM containing 10% (v/v) FBS, 1% nonessential amino acids, 100 U/mL penicillin G, and 100 μg/ml streptomycin, and incubated at 37°C in the presence of 5% CO_2_ and 95% humidity. The Neuro-2a-CYP46A1 cells were seeded into six-well plates at a density of 1 × 10^5^ cells per well and cultured for 24 h. Drug intervention experiments were conducted by replacing the medium with a serum-free medium containing sesame ligands (20 μM) for 24 h.

### Animal studies

The experiments were reviewed and approved by the Experimental Animal Centre of Dalian Medical University. All rats were obtained from Liaoning Changsheng Biotechnology co., Ltd. All animal experiments complied with the ARRIVE guidelines and were carried out in accordance with the National Institutes of Health guide for the care and use of laboratory animals (NIH Publications No. 8023, revised 1978). Wistar rats were randomly divided into the administration group (n = 5) and the control group (n = 5). The rats were provided with laboratory standard animal feed and kept under a 12-h light cycle. Sesamin (60 mg/kg) was given by intragastric administration daily for 4 weeks; the control group was given an equivalent volume of olive oil. The rats were anesthetized with pentobarbital sodium (40 mg/kg) and blood samples and brain tissue samples were collected for further assays.

### Western blotting analysis

The cells and tissues were harvested with lysis buffer and cleaved at 4°C for 20 min. All samples were centrifuged at 12,000 × g for 15 min at 4°C. The protein concentrations were quantified by BCA regent kits. Sodium dodecyl sulfate-polyacrylamide gel electrophoresis (SDS-PAGE) was used to separate the proteins, and they were then transferred to PVDF membranes. After incubation of the membranes with 5% non-fat milk for 2 h, they were then incubated with the primary antibodies at 4°C overnight. After washing three times, the membranes were incubated with the secondary antibody for 2 h. The protein bands were detected by a Tanon-5200 imaging system and the densities were analyzed by ImageJ software. The GAPDH antibody served as the internal control for sample loading.

### Detection of 24*S*-hydroxycholesterol

The levels of 24*S*-hydroxycholesterol in rat plasma and brain tissues were measured by ELISA kits (Proteintech Group, Wuhan, China), according to the manufacturer’s instructions.

### Quantitative real-time PCR

The experimental procedure was performed as described previously (Wen et al., 2018). Total brain RNA and cDNA synthesis were meausured with an RNAiso Plus Reagent Kit and SYBR Premix Ex Taq Kit, respectively (Takara Biotechnology, Dalian, China). Quantitative real-time PCR was performed using SYBR Green PCR Master Mix and an ABI prim 7500 Sequence Detection System (Applied Biosystems, United States). Primers used for RT-qPCR are shown in Supplementary Table S2.

### Statistical analysis

The data were analyzed by one-way ANOVA and unpaired t-tests using GraphPad Prism software (Version 7.0, GraphPad); *p* < 0.05 was considered statistically significant.

## Results

### Inhibition assays of CYP46A1 activity

**FIGURE 2:**
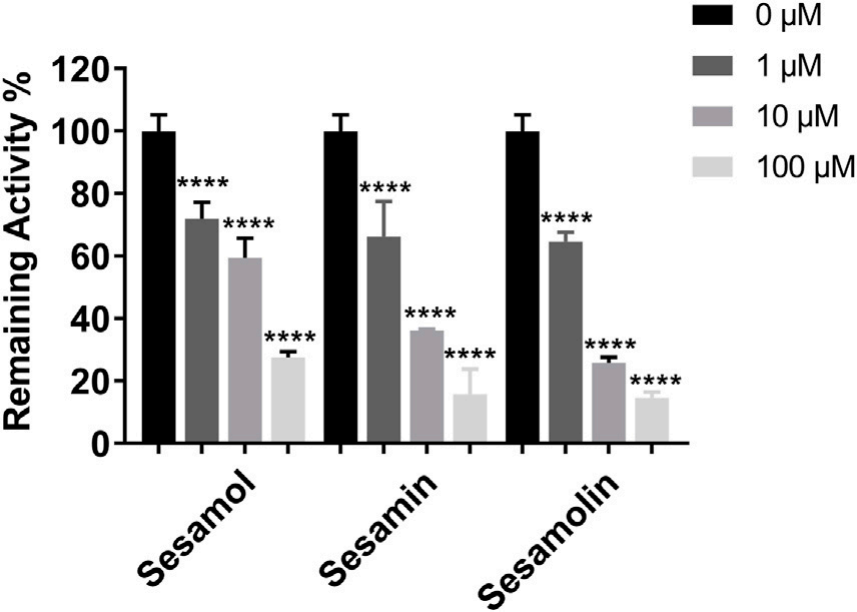
The inhibitory effects of sesame lignans on human CYP46A1 were determined using testosterone as the substrate probe. Testosterone (10 μM) was incubated with human CYP46A1 at 37°C in the absence and presence of sesame lignans (0, 1, 10, and 100 μM). Data were analyzed by one-way ANOVA and unpaired t-tests and are presented as mean ± SD from three experiments carried out in duplicate. ****p < 0.0001 compared with control.

**FIGURE 3:**
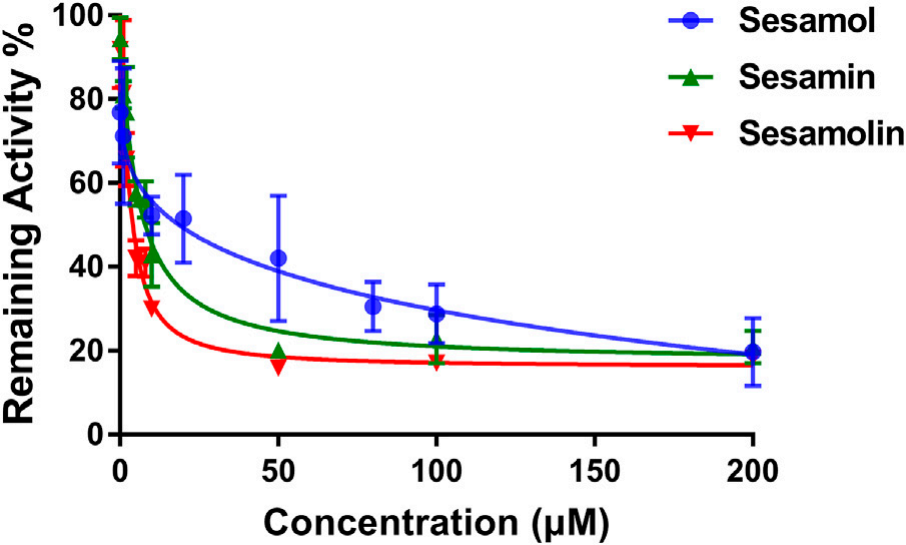
Dose-response inhibition curves of sesamol, sesamin, and sesamolin against human CYP46A1 using testosterone as the substrate. Results were shown as the mean ± SD of three experiments.

**FIGURE 4:**
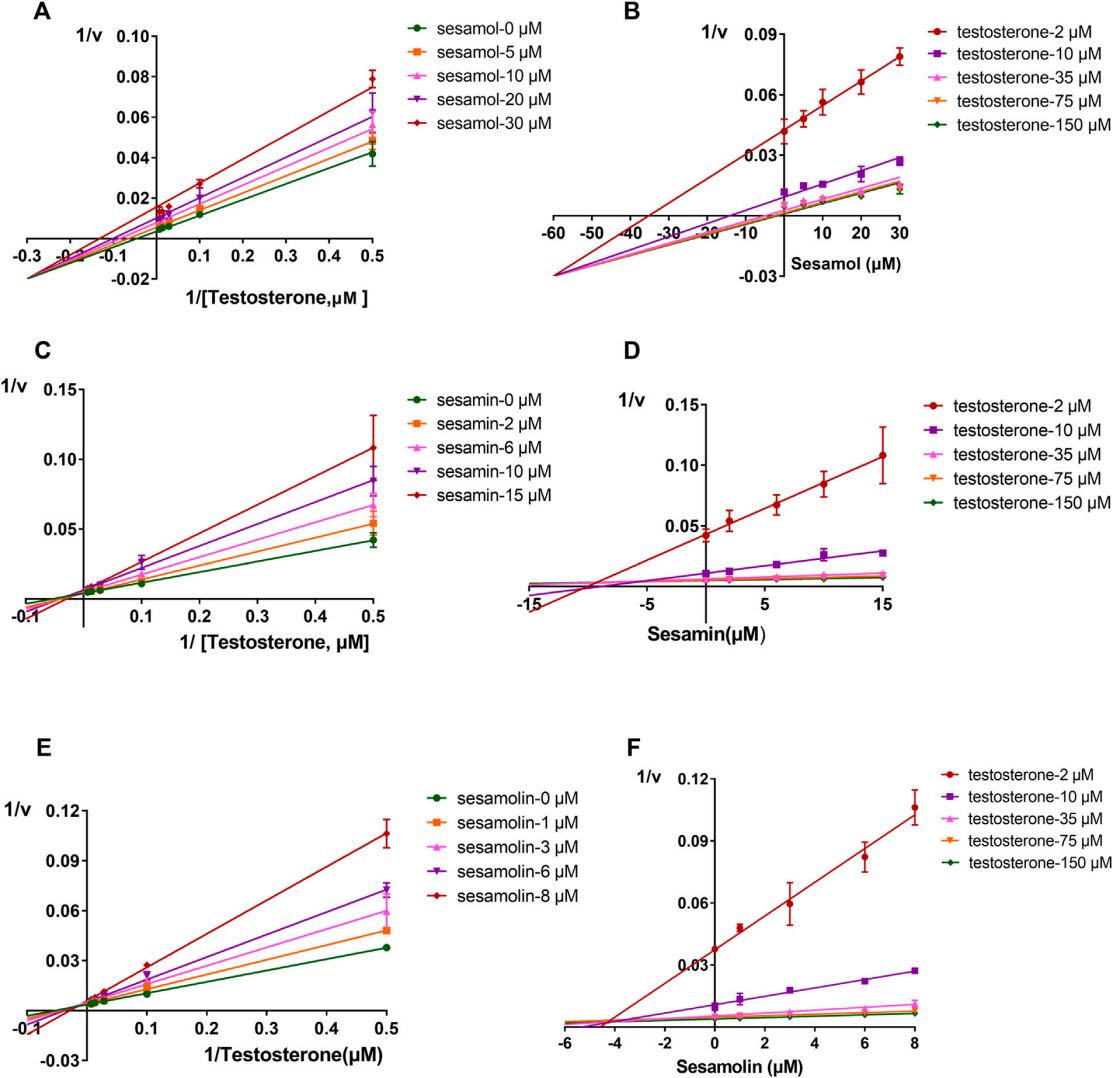
Lineweaver-Burk plots **(A,C, and (E))** and Dixon plots **(B,D, and (F))** for the inhibition effects of sesamol, sesamin, and sesamolin against CYP46A1 activity using testosterone as the substrate **(B,D, and (F))**. Data points were from three independent experiments.

The inhibitory effects of sesame lignans on CYP46A1 were studied using testosterone as a substrate. Sesame lignans at concentrations of 0, 1, 10, and 100 μM were selected to preliminarily screen the inhibitory potential of CYP46A1. When the concentrations of sesamol, sesamin, and sesamolin were 10 μM, the residual activities of CYP46A1 were 59.4%, 36.2%, and 25.8%, respectively. (Figure 2). To further compare the inhibitory ability of the three compounds on CYP46A1 enzyme activity, the half-maximal inhibition concentration (IC_50_) of each compound on CYP46A1 enzyme activity was determined. As shown in Figure 3, sesamin and sesamolin showed similar inhibitory intensity on CYP46A1, with IC50 values of 6.016 and 3.34 μM, respectively, while sesamol had a relatively weak inhibitory intensity, with an IC50 value of 21.3 μM. To further explore the inhibitory mechanism of these sesame lignans, an inhibitory kinetic study was conducted. Lineweaver-Burk plots (Figures 4A, C, E) and related Dixon plots (Figures4B, D, F) were drawn using double-reciprocal nonlinear regression analysis. Sesamin (Figures 4C, D) and sesamolin (Figures 4E, F) exhibited mixed inhibition, with inhibition constant (*K*i) values of 6.87 and 4.02 μM, respectively. Sesamol (Figures 4A, B) showed non-competitive inhibitory behavior, with a Ki value of 18.7 μM. The inhibitory kinetic parameters of the sesame lignans on CYP46A1 are shown in Table 1.

**TABLE 1:** The inhibitory kinetic parameters of sesame lignans on CYP46A1.

Compound	IC_50_ (μM)	Ki (μM)	Type of inhibition	α	*R* ^2^
Sesamol	21.3 ± 4.739	18.7 ± 0.720	Non-competitive	—	0.985
Sesamin	6.02 ± 0.966	6.87 ± 1.065	Mixed	6.491	0.9851
Sesamolin	3.34 ± 0.543	4.02 ± 0.786	Mixed	4.891	0.9701

### Molecular docking simulation

**FIGURE 5:**
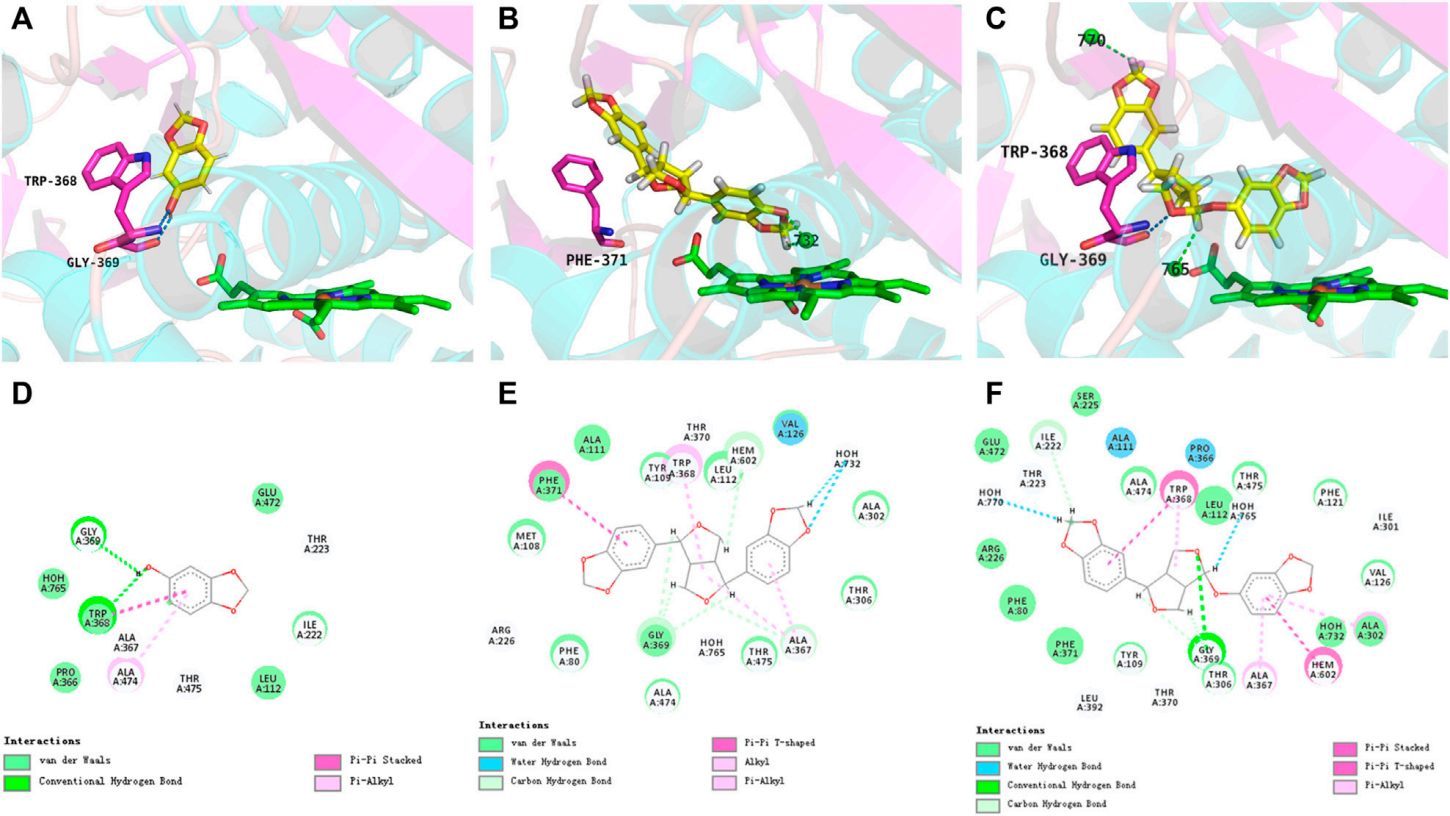
Views of the CYP46A1 active site illustrating interactions with sesamol **(A)**, sesamin **(B)**, and sesamolin **(C)**. The heme iron and water molecules (W770, W765, and W732) are represented by brown and green spheres, respectively. Dashed blue and green lines indicate conventional hydrogen bonds and water-hydrogen bonds, respectively. The interacting 2D plots of sesamol **(D)**, sesamin **(E)**, and sesamolin **(F)** located within the active site of CYP46A1.

Molecular docking simulations were performed to further explore the molecular interactions between the sesame lignans and the CYP46A1 enzyme. The optimal orientations of sesamol, sesamin, and sesamolin in CYP46A1 were shown in Figure 5, with total scores of 3.17, 6.16, and 6.75, respectively. Notably, the total scores of sesamin and sesamolin were significantly higher than that of sesamol, which is in accordance with their inhibitory activities. The enzyme-ligand interactions within the CYP46A1 active site mainly involved a conventional hydrogen bond, π−π stack, and classical water hydrogen bond. Sesamolin formed a hydrogen bond with Gly369 and a π−π stacking interaction with Trp368; sesamin formed a π−π stacking interaction with Phe371; sesamol formed two hydrogen bonds with Gly369 and Ttp368 and a π−π stacking interaction with Trp368. These residues interacted with the sesame lignans to form a network of hydrogen bonds, which facilitated the binding of the sesame lignans with the active site of CYP46A1. Notably, sesamolin formed two water-hydrogen bonds with W765 and W770 and sesamin formed two water-hydrogen bonds with W732, which were located near the heme. These results suggest that the sesame ligands have marked binding affinity with CYP46A1, which is in accordance with their inhibition effects. In addition, the interaction between CYP46A1 and soticlestat was also simulated. The optimal orientation of soticlestat was shown in Supplementary Figure S1, with a total score of 6.74, a relatively high ChemScore of −38.311 and a relatively low CScore of 2. Soticlestat formed two hydrogen bonds with Gly369 and Trp 368, one classical water hydrogen bond with W765, and two π−π stacking interactions with Trp368. The molecular docking parameters of the sesame lignans are shown in Supplementary Table S3.

### Membrane permeability

**FIGURE 6:**
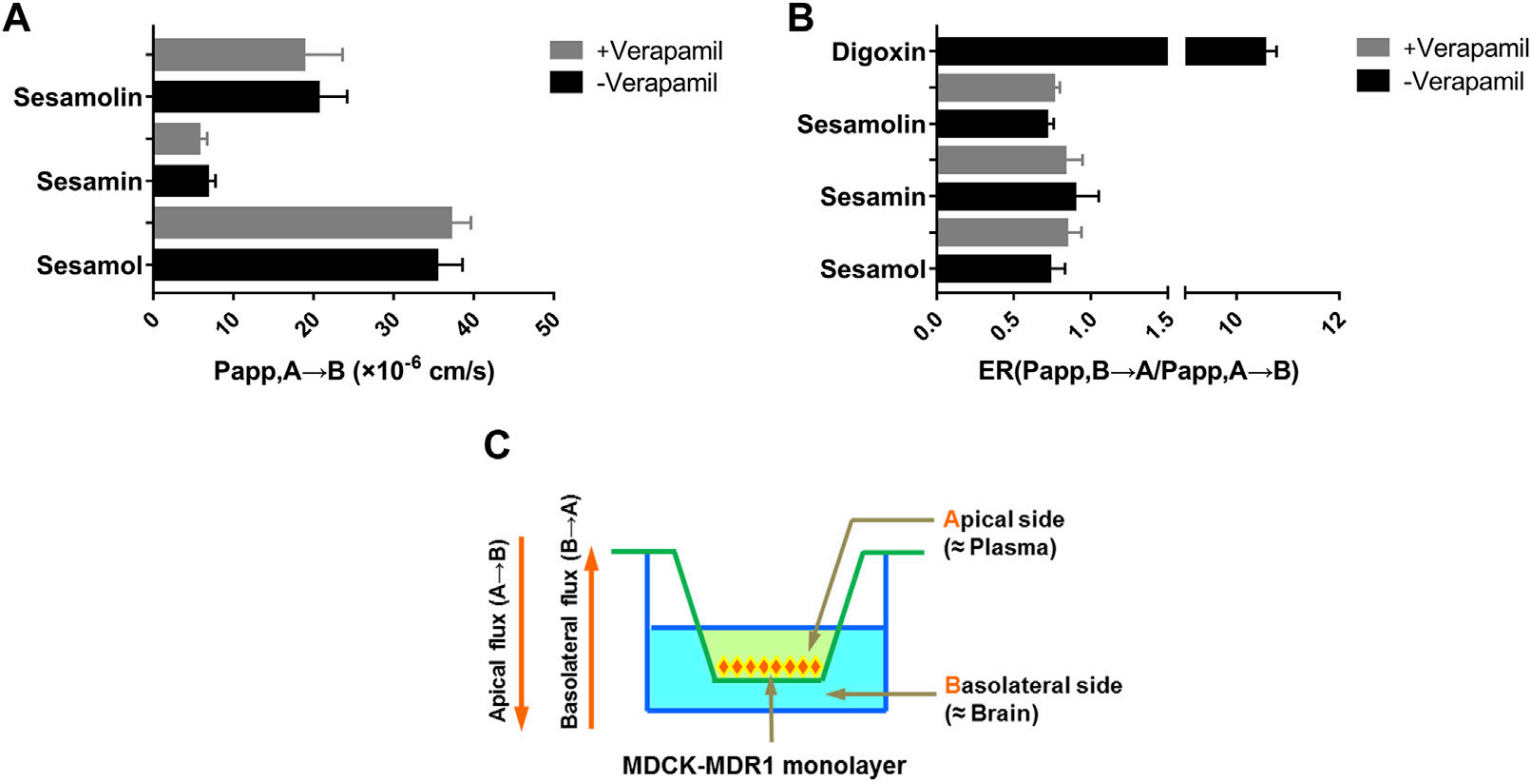
Membrane permeability **(A)** and efflux ratio **(B)** of sesamin, sesamolin, and sesamol across the MDCK-MDR1 monolayer **(C)**. Data are expressed as the mean ± SD (*n* = 3).

Digoxin, a P-gp substrate, was first utilized as a probe drug to confirm the transmembrane transport mechanism. The transport of digoxin from the basal to the apical side was significantly greater than the opposite direction in MDR1-MDCK cells with ER values of 10.5, indicating a strong efflux transport mediated by P-gp. The result suggested that the transport model had been established effectively. The membrane permeabilities of sesamol, sesamin, and sesamolin across the MDCK-MDR1 monolayer compounds are shown in Figure 6. Overall, the three compounds exhibited high permeability coefficients (*P*_app_ > 1 × 10^–6^ cm/s). The *P*_app_ values of sesamol, sesamin, and sesamolin were 35.32, 6.75, and 20.53 × 10^–6^ cm/s, respectively. Notably, their efflux ratios (*P*_app, B→A_/*P*_app, A→B_) were less than one and the ER values did not decrease to <50% of the ER in the absence of verapamil, which suggests that they are not substrates of efflux transporters, particularly P-gp, in MDCK-MDR1 cells. However, some uptake transporters might mediate their transmembrane transport. These results suggest that the sesame lignans are easily absorbed and distributed in brain tissue.

### Effects of sesame lignans on CYP46A1 protein expression in h-CYP46A1-Neuro-2a

**FIGURE 7:**
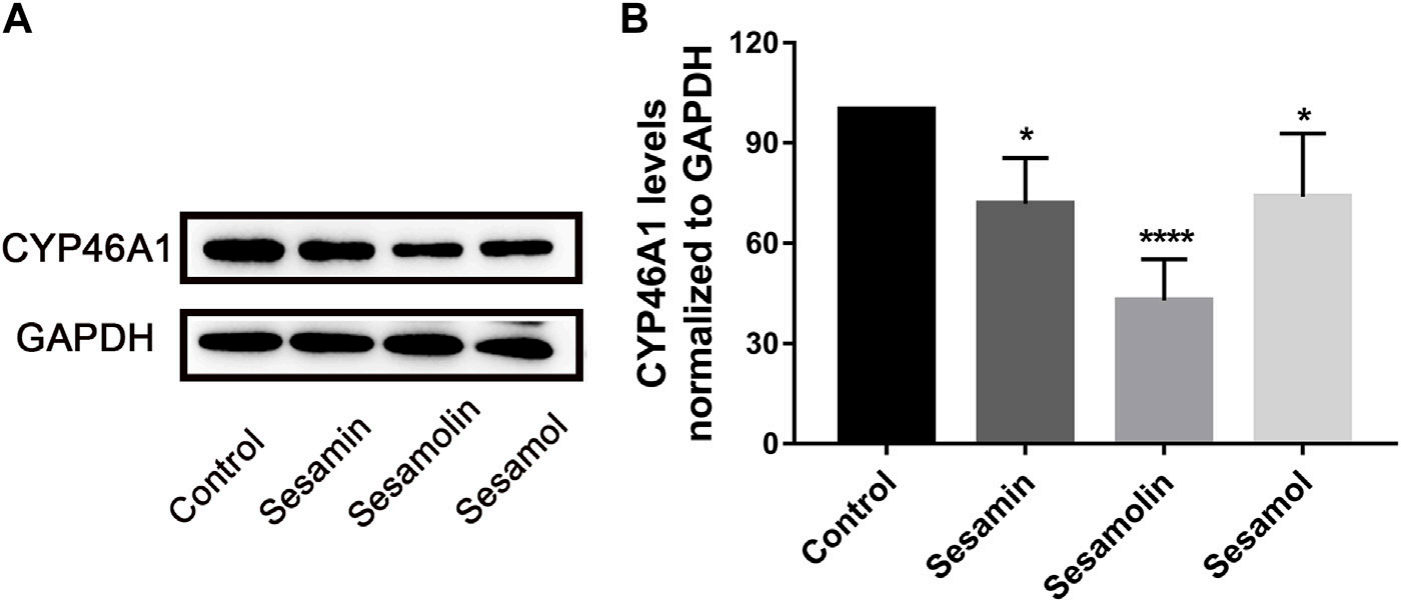
Effects of sesamin, sesamolin, and sesamol on protein expression of **(A)** and statistical analysis **(B)** of CYP46A1 in h-CYP46A1-Neuro-2a. Data were analyzed by one-way ANOVA and unpaired t-tests and are presented as mean ± SD from five experiments carried out in duplicate. *p < 0.05, ****p < 0.0001 compared with control.

To evaluate the effects of sesamin, sesamolin, and sesamol on CYP46A1 content in h-CYP46A1-Neuro-2a, the cell lysate was subjected to Western blotting. Compared with the control group, treatment with sesamin, sesamolin, and sesamol significantly reduced CYP46A1 protein levels by 28.1% (*p* < 0.05), 57.1% (*p* < 0.0001), and 26.1% (*p* < 0.05), respectively (Figure 7).

### Changes of CYP46A1 activity *in vivo* after long-term exposure to sesamin

**FIGURE 8:**
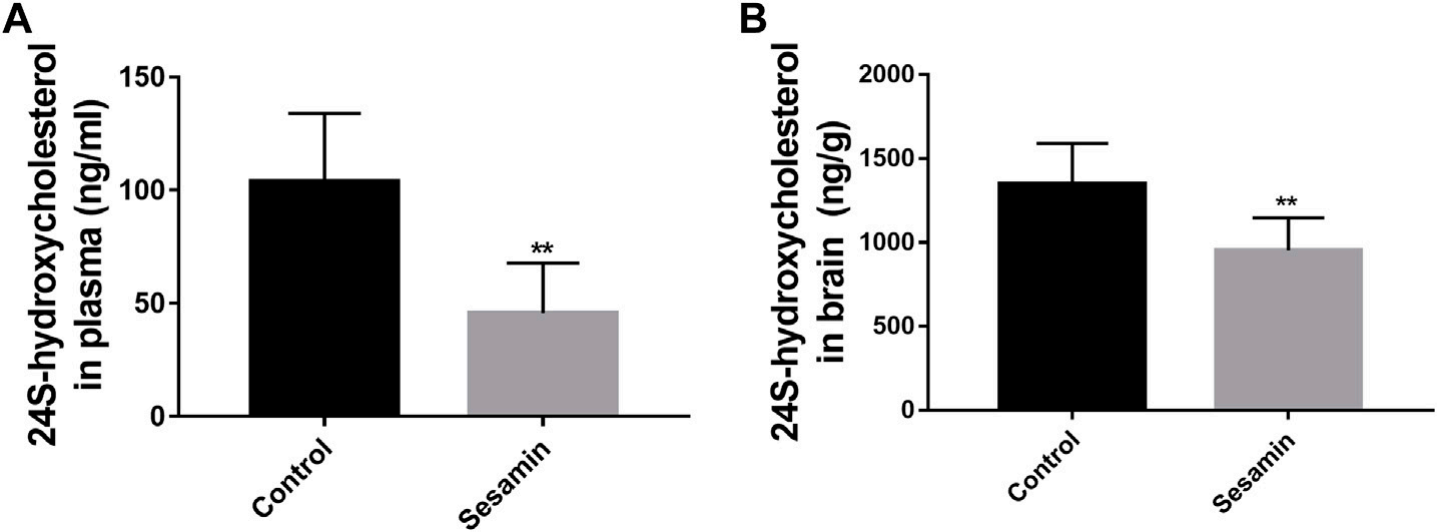
Changes in the levels of 24S-hydroxycholesterol in rat plasma **(A)** and brain tissues **(B)** after treatment with sesamin. Data were analyzed by one-way ANOVA and unpaired t-tests and are presented as mean ± SD from five experiments carried out in duplicate. **p < 0.01 compared with control.

To assess the effect of sesamin on the activity of CYP46A1 *in vivo*, rats received intragastric administration of sesamin (60 mg/kg) once a day for 4 weeks. As shown in Figure 8B, the level of 24*S*-HC in the brain was significantly decreased by 29.3% (*p* < 0.01). Most of the circulating 24*S*-HC comes from the brain, so a reduction in brain synthesis reduces 24*S*-HC levels in plasma. The plasma concentration of 24*S*-HC in the control group was 103.69 ng/ml. In contrast, the plasma concentration of 24*S*-HC in rats treated with sesamin was 45.747 ng/ml (Figure 8A). The level of 24*S*-HC in plasma was significantly reduced by 55.88% (*p* < 0.01).

### Influence of sesamin on the expressions of CYP46A1, NMDAR2A, NMDAR2B, and HMGCR in the rat brain

**FIGURE 9:**
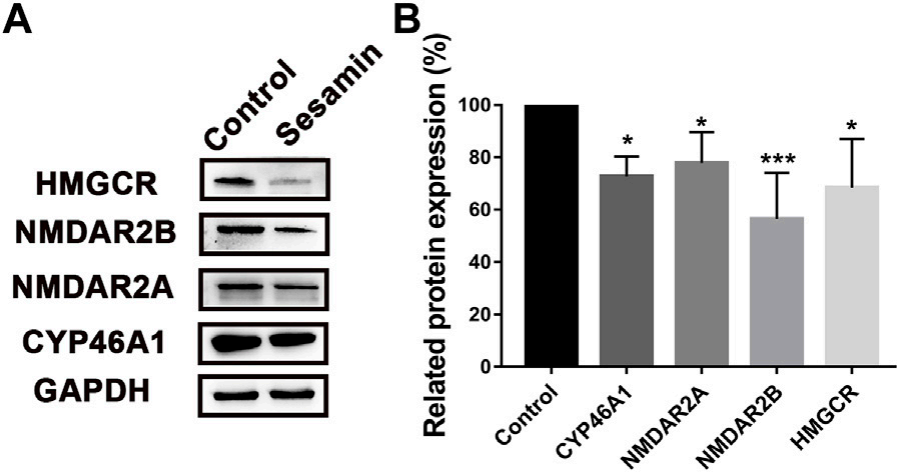
Protein expression changes **(A)** and statistical analysis **(B)** in CYP46A1, NMDAR2A, NMDAR2B, and HMG-CoA in rat brain tissues after treatment with sesamin. Data were analyzed by one-way ANOVA and unpaired t-tests and are presented as mean ± SD from five experiments carried out in duplicate. *p < 0.05, ***p < 0.001 compared with control.

**FIGURE 10:**
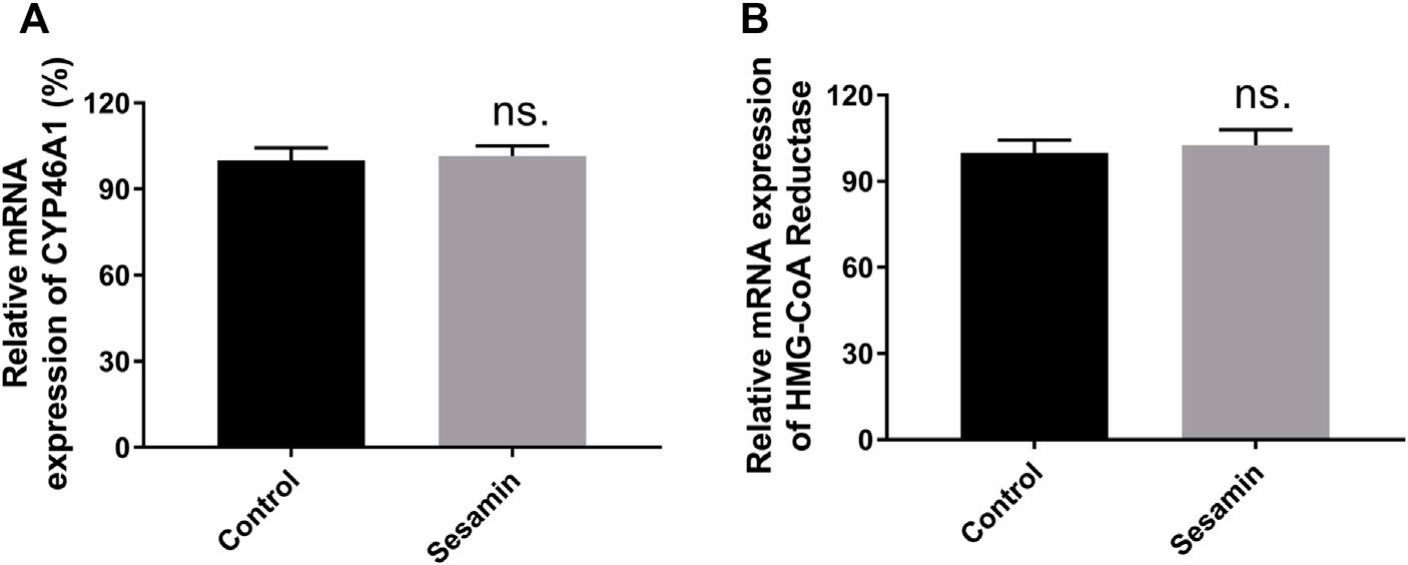
mRNA expression changes in CYP46A1 **(A)** and HMG-CoA Reductase **(B)** mRNA in rat brain tissues after treatment with sesamin. Data were analyzed by one-way ANOVA and unpaired t-tests and are presented as mean ± SD from five experiments carried out in duplicate. ns: not significant (*p* > 0.05 compared with control).

The regulatory effects of sesamin on CYP46A1, NMDAR2A, NMDAR2B, and HMGCR protein levels were analyzed using Western blotting. After 4 weeks of treatment with sesamin, the protein expression levels of CYP46A1 and HMGCR in rat brain tissue were significantly reduced by 27.3% (*p* < 0.05) and 31.6% (*p* < 0.05), respectively, compared with the control group (Figure 9). In contrast, the mRNA expression levels of CYP46A1 and HMGCR in the sesamin-treated group exhibited no significant changes compared with the control group (Figure 10). Notably, the protein expression levels of NMDAR2A and NMDAR2B in rat brain tissue were significantly reduced by 22.1% (*p* < 0.05) and 43.5% (*p* < 0.001), respectively, in the sesamin-treated group compared with the control group (Figure 9).

## Discussion and conclusion

The present study evaluated the effects of three sesame lignans on CYP46A1 *in vitro* and *in vivo*. *In vitro* inhibition kinetics studies indicated that sesamin and sesamolin exhibit strong mixed partial competitive inhibition of CYP46A1, with similar Ki values of 6.87 and 4.02 μM, respectively. The docking simulations showed that the sesame lignans favored binding to CYP46A1 when forming water-hydrogen bonds with W732, W765, and W770, which are located near the heme of CYP46A1. The high membrane permeability suggests that sesamin lignans are easily absorbed and distributed in brain tissue. In the *in vivo* experiments, sesamin reduced the content of 24*S*-HC both in the brain and plasma, decreased the expression of the CYP46A1 protein but did not affect mRNA expression, and decreased the expression of the NMDAR2A, NMDAR2B, and HMGCR proteins. The inhibitory effect of sesamin on CYP46A1 may contribute to a treatment for neural hyperexcitation diseases such as epilepsy.

The three sesame lignans all have Methylenedioxyphenyl (MDP) structures; MDP compounds interact with mammalian CYP enzymes in a variety of ways to inhibit or promote CYP enzyme activity (Murray, 2000). Sesamin inhibits CYP3A4 activity and reduces its protein expression by antagonizing the activation of progesterone X receptor (Lim et al., 2012). In addition, sesamin competitively inhibits the 7-position hydroxylation of S-warfarin mediated by CYP2C9, with a value of 13.1 μM (Fujii et al., 2020). It has been reported that sesamin reduces 20-hydroxyeicosatetraenoic acid (20-HETE) in human plasma and urine by inhibiting CYP4F2, a human subtype of an enzyme that synthesizes 20-HETE from arachidonic acid (J. Wu et al., 2009). Thus, the MDP moiety of sesamin lignans may be the key structural feature enabling the inhibition of CYP46A1.

In this study, sesamin inhibited the expression of the CYP46A1 protein in the rat brain but did not affect CYP46A1 mRNA. The promoter region of the human CYP46A1 gene, which has a high content of guanine and cytosine (GC), has no typical thymine-adenine-thymine-adenine (TATA) or cytosine-adenine-adenine-thymine (CAAT) boxes, according to transcriptional regulation studies. Aside from the increase in transcriptional activity produced by oxidative stress, researchers have not yet identified a treatment that can significantly alter the transcriptional activity of the CYP46A1 promoter structure (Ohyama et al., 2006). The findings of the current study may be explained by the fact that sesamin does not alter CYP46A1 gene transcription but may play a role in post-translational modification and other processes. Alternatively, binding with sesamin may promote protein breakdown, resulting in lower CYP46A1 protein expression.

N-methyl-d-aspartate receptors (NMDARs) are among the primary excitatory receptors on synapses, regulating the balance between neuronal excitation and inhibition (Hanada, 2020). NMDARs are ionotropic glutamate receptors located in the brain and are involved in neuroplasticity, excitatory neurotransmission, and neurotoxicity (Horak et al., 2021). Previous studies have shown that over-activation of NMDARs leads to nerve cell death in neurological diseases such as epilepsy, stroke, PD, and AD (Essiz et al., 2021). Notably, glutamate overactivates NMDAR and increases the generation of ROS, which causes oxidative stress and oxidative cell death (Quincozes-Santos et al., 2014). Exogenous 24*S*-HC aggravates NMDAR-dependent excitatory toxicity in primary neuron culture after being subjected to oxygen-glucose deprivation (OGD), as well as reduced endogenous 24*S*-HC synthesis alleviates OGD-induced cell death (Sun et al., 2017). Besides the excitatory toxicity, 24*S*-HC has also been identified as a powerful inducer of oxiapoptophagy, which involves simultaneous oxidative stress, apoptosis, and autophagy (Nury et al., 2015) (Gamba et al., 2011). (Nury et al., 2015). In this study, sesamin significantly inhibited the acticity of CYP46A1 *in vitro* and reduced the 24*S*-HC level *in vivo*, as well as down-regulated the expression levels of the NMDAR2A and NMDAR2B proteins in rat brain tissue. These findings strongly suggest that sesamin has therapeutic potential for neural hyperexcitation diseases.

In conclusion, naturally occurring sesame lignans exhibited remarkable inhibitory action against CYP46A1 *in vitro* and *in vivo*. Molecular simulations revealed that sesamin and sesamolin had excellent orientations within CYP46A1 by forming two water-hydrogen bonds, located near the heme of CYP46A1, which were consistent with their inhibitory intensities towards CYP46A1. Notably, the sesame lignans also had high permeability, implying that they may be easily absorbed and distributed in the brain. More importantly, sesamin dramatically lowered 24*S*-HC levels *in vivo* and lowered the protein expression levels of CYP46A1, NMDAR2A, NMDAR2B, and HMGCR in the brain tissues. Furthermore, These findings may help the development of effective therapies for neurological illnesses *via* modulation of CYP46A1 activity and provide a basis for further exploration of the therapeutic possibilities of targeting CYP46A1.

## Data Availability

The raw data supporting the conclusions of this article will be made available by the authors, without undue reservation.
