# Supplementary material for: Characterization of three naturally occurring lignans, sesamol, sesamolin, and sesamin, as potent inhibitors of human cytochrome P450 46A1: Implications for treating excitatory neurotoxicity
Source: Front Pharmacol. 2022 Nov 22;13:1046814. doi: 10.3389/fphar.2022.1046814 (PMC9722955; doi:10.3389/fphar.2022.1046814)
Supplement: Supplementary file 1 [file DataSheet1.pdf]

Characterization of three naturally occurring lignans, sesamol, sesamolin, and sesamin, as potent inhibitors of human cytochrome P450 46A1: Implications for treating excitatory neurotoxicity

Jie Du,[1,2#] Xiaodong Chen,[1,2#] Yongshun Zhao,[3#] Tingting Zhao,[1,2] Dalong Wang,[1,2] Zujia Chen,[1,2] Changyuan Wang,[1,2] Qiang Meng,[1,2] Jialin Yao,[1,2], Huijun Sun,[1,2] Kexin Liu,[1,2] and Jingjing Wu [1,2*]

Affiliations:

1 College of Pharmacy, Dalian Medical University, Dalian, China.

2 Provincial Key Laboratory for Pharmacokinetics and Transport, Liaoning Dalian Medical University, Dalian, China.

3 The First Affiliated Hospital, Dalian Medical University, Dalian, China.

# These authors contributed equally to this work.

* **Correspondence:**

Dr. Jingjing Wu

ORCID: 0000-0002-5046-9996

Department of Clinical Pharmacology, College of pharmacy

Dalian Medical University, Dalian, China.

Supplementary Tables

Table S1 MS/MS conditions for analyzed compounds

analyte	transition (*m/z*)	DP (eV)	CE (eV)	CEP (eV)	EP (eV)	CXP (eV)
16β-hydroxytestosterone	305.2→97.2	59.0	35.0	18.65	7.0	8.0
Carbamazepine	370.4→288.1	60.0	42.0	16.40	10.0	14.0

Table S2 Primers used for RT-qPCR

Gene	Forward 5′-3′	Reverse 5′-3′
CYP46A1	AGCATAGGAGGCTACAACG	CTGCCATCAAGGACAACT
HMGCR	CTGCATCGGGCAGCAGTT	CAGTGGCTTGAGCGTAGCC
β-actin	TATTGGCAACGAGCGGTTC	ATGCCACAGGATTCCATACCC

Table S3 Molecular docking simulation of compounds with sesamol, sesamin, sesamolin, and soticlestat

Compounds	Total Score	Chem Score	CScore	Enzyme-ligand interactions		
				Conventional Hydrogen Bond	Classical Water Hydrogen Bond	Pi-Pi stack
Sesamol	3.17	-14.745	4	Gly369, Trp368	-	Trp368
Sesamin	6.16	-27.53	5	-	W732 (n=2)	Phe371
Sesamolin	6.75	-24.393	5	Gly369	W765, W770	Trp368
Soticlestat	6.74	-38.311	2	Gly369, Trp368	W765	Trp368 (n=2)

Supplementary Figure

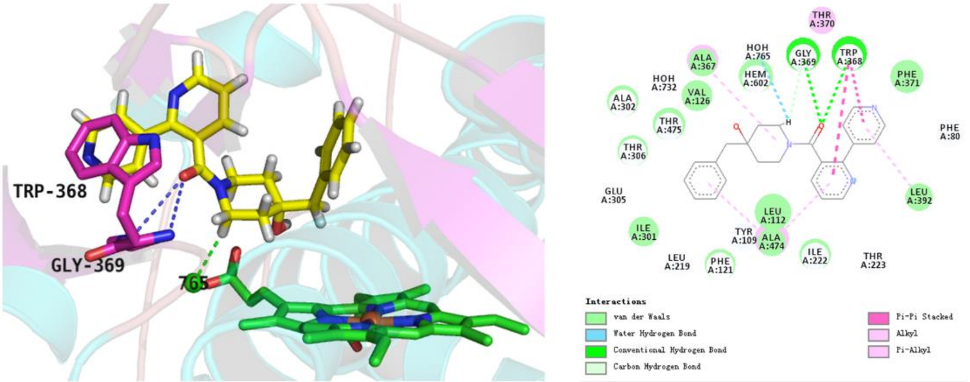

Fig. S1 Molecule docking simulations of soticlestat with CYP46A1

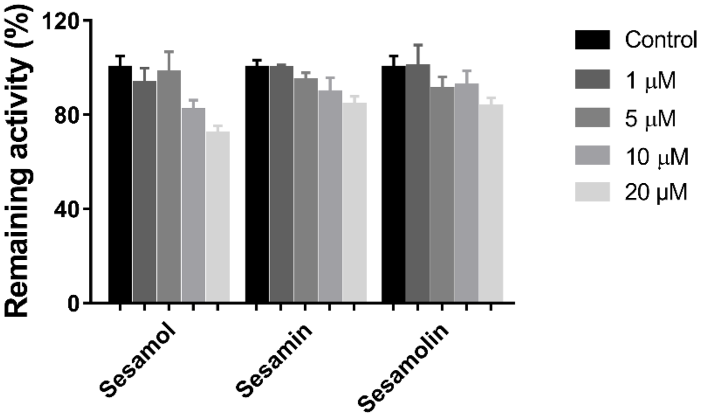

Fig. S2 Cell viability after treatment with various concentrations sesame lignans
